# Integrated analysis of genomic and transcriptomic profiles identified a prognostic immunohistochemistry panel for esophageal squamous cell cancer

**DOI:** 10.1002/cam4.2744

**Published:** 2019-12-02

**Authors:** Yue Yu, Zhihua Li, Chenjun Huang, Haisheng Fang, Fei Zhao, Yue Zhou, Xianglong Pan, Qifan Li, Yu Zhuang, Liang Chen, Jing Xu, Wei Wang

**Affiliations:** ^1^ Department of Thoracic Surgery The First Affiliated Hospital of Nanjing Medical University Nanjing China; ^2^ Department of Thoracic Surgery Chinese Academy of Medical Sciences Cancer Institute and Hospital Beijing China; ^3^ Department of Epidemiology and Biostatistics Jiangsu Key Lab of Cancer Biomarkers, Prevention and Treatment Collaborative Innovation Center for Cancer Personalized Medicine School of Public Health Nanjing Medical University Nanjing China; ^4^ Department of Pathology The First Affiliated Hospital of Nanjing Medical University Nanjing China

**Keywords:** *ANO1*, bioinformatics, esophageal squamous cell cancer, *MMP3*, prognosis

## Abstract

**Background:**

The poor outcome of patients with esophageal squamous cell carcinoma (ESCC) highlights the importance of the identification of novel effective prognostic biomarkers. We aimed to identify a clinically applicable prognostic immunohistochemistry (IHC) panel for ESCC.

**Methods:**

An integrated analysis was performed to screen and establish a prognostic panel using exome sequencing profile from 81 pairs of ESCC samples and RNA expression microarray data from 119 ESCC subjects. Two independent ESCC cohorts were recruited as training and validation groups to test the prognostic value.

**Results:**

Three genes were selected, namely, *ANO1*, *GAL,* and *MMP3*, which were aberrantly expressed in ESCC tumor tissues (*P* < .001). Among them, *ANO1* and *MMP3* were reserved for the construction of the prognostic panel due to their significant association with the prognosis of ESCC patients (*P* = .015 and *P* < .001). Patients with both *ANO1*+ and *MMP3*+ had a poorer prognosis than that with *ANO1*−/*MMP3*+, *ANO1*+/*MMP3*−, or *ANO1*−/*MMP3* − in both the training set and validation set (*P* < .001). Receiver operating characteristic analysis showed that the combination of IHC panel and eighth American Joint Commission on Cancer staging yielded a better prognostic predictive efficacy compared with the two indexes alone (*P* < .001, area under curve: 0.752). Finally, a nomogram was created by integrating the IHC markers and clinicopathological risk factors to predict prognosis with a C‐index of 0.695 (95% confidence interval: 0.657‐0.734).

**Conclusion:**

Using an integrated multistage screening strategy, we identified and validated a valuable prognostic IHC panel for ESCC.

## Graphical abstract

Using an integrated analysis of genomic and transcriptomic profiles from esophageal squamous cell carcinoma patients, we had identified and a valuable immunohistochemistry panel comprising *ANO1* and *MMP3*. Patients could be stratified into three subgroups with different risks based on the positive marker status of two genes. In addition, we constructed an immunohistochemistry‐based nomogram, which provides improved risk stratification and might be a prognostic tool for facilitating clinical management of treatment.

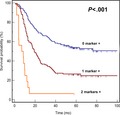

## 1 INTRODUCTION

Esophageal cancer has its highest prevalence in China and is ranked third for incidence and fourth for mortality.^1^, ^2^ Approximately 70% of global esophageal cancer cases occur in China, with esophageal squamous cell carcinoma (ESCC) being the most common histopathological form, accounting for more than 90% of esophageal cancer cases.^3^ Despite advances in clinical diagnosis and treatment, the 5‐year overall survival (OS) of ESCC ranges from 15% to 25%.^4^

Accurate assessment for the prognosis of ESCC patients is crucial to guide clinical management and to further improve the clinical outcome. The American Joint Commission on Cancer (AJCC) TNM (tumor, node, and metastasis) staging classification is the key determinant for prognostic prediction and risk stratification for treatment decisions, which takes into account the depth of tumor invasion, nodal status, and metastatic disease.^5^ However, the AJCC staging system is not sufficient to predict the outcome of ESCC patients without considering the biology or molecular features of each individual tumor.^6^, ^7^ Therefore, identification of the key prognostic biomarkers, as effective survival predictors and therapeutic targets, is highly important for the current clinical management of ESCC.^8^, ^9^

Protein is the ultimate performer of multiple biological functions, and the relationships between abnormal expressed proteins and cancer have been widely studied.^6^, ^10^ Previous studies have reported the vital effect of multiple somatic genetic alterations in the development of cancers, in which copy number variations (CNVs) of DNA are closely related to abnormal expression of protein and can be used for the selection of prognostic biomarkers in malignant tumors.^11^, ^12^ In addition, other studies have aimed to identify potential prognostic proteins based on altered transcriptomic levels.^13^, ^14^ All these studies have suggested that both genomic and transcriptomic profiles may provide valuable information for the prediction of ESCC prognosis.

However, few papers have investigated prognostic protein markers based on the integrated analysis of genomic and transcriptomic profiles.^15^ In this study, we performed an integrated analysis on both somatic CNVs and differently expressed mRNAs. The whole‐exome sequencing from 81 paired ESCC samples^9^ and RNA expression microarray^16^ data from 119 pairs of ESCC patients were used to screen the prognostic biomarker candidates. Furthermore, two independent ESCC cohorts, including 197 subjects in the training set and 118 samples in the validation set, were recruited to determine the final biomarkers. Finally, the prognostic model for ESCC was constructed, and a nomogram was depicted. This study established an optimized panel of immunohistochemical (IHC) markers that can be used to segregate ESCC patients into different prognostic subgroups.

## 2 MATERIAL AND METHODS

### 2.1 Patients and tissues

This study was approved by the Medical Ethics Committees of the First Affiliated Hospital of Nanjing Medical University/Jiangsu Province Hospital and Chinese Academy of Medical Sciences Cancer Institute and Hospital. All procedures were in accordance with the ethical standards of the Responsible Committee on Human Experimentation (institutional and national) and with the Helsinki Declaration of 1964 and later versions. Informed consent or substitute for it was obtained from all patients included in the study.

Two independent patient sets were recruited from North and South China for the training and validation groups, respectively. The cohort consisted of 197 patients with ESCC who received surgery in North China from the Chinese Academy of Medical Sciences Cancer Institute and Hospital (CAMS set), Beijing, between January 2005 and December 2007, and this cohort was used to establish the prognostic IHC panel. For validating the IHC panel, 118 cases with ESCC who were treated at the First Affiliated Hospital of Nanjing Medical University/Jiangsu Province Hospital (JSPH set), Nanjing between January 2002 and December 2003 were enrolled as the independent validation set in South China.

The inclusion criteria were as follows: (a) definitive diagnosis of esophageal cancer by preoperative electronic gastroscopy with biopsy, barium meal, and enhanced computed tomography of the chest and upper abdomen; (b) pathological type of ESCC by biopsy; and (c) adequate pulmonary function allowing the use of single‐lung ventilation. The exclusion criteria were as follows: (a) history of gastric resection; (b) history of chest surgery; (c) neoadjuvant chemotherapy and/or radiotherapy; (d) distant metastasis; (e) impaired cardiac, kidney or liver function; (f) impaired coagulation; or (g) palliative resection or positive margin. Disease stages were classified based on the eighth edition AJCC Staging Manual defined as pathological TNM stages (5).

### 2.2 Tissue microarray construction and immunohistochemistry

Tissue microarrays were prepared from archival formalin‐fixed, paraffin‐embedded tissue blocks (RaiseDragon Co., Ltd. Beijing). For each tumor, a representative tumor area was carefully selected from a hematoxylin‐ and eosin‐stained section. For each case, normal tissue and cancer tests were repeated twice. The training and validation cohort samples were placed on different tissue microarray sections.

The avidin‐biotin complex method was used for IHC analysis. Briefly, after deparaffinization, slides were rehydrated in decreasing concentrations of ethanol and rinsed in phosphate‐buffered saline (PBS). Sections were then subjected to an antigen retrieval process. After rinsing in PBS, endogenous peroxidase was inactivated by 3% hydrogen peroxide, and nonspecific‐binding sites were blocked by incubation in 10% normal animal serum. Sections were incubated at 4°C for 24 h with primary antibodies against *ANO1* (RMA‐0610, Maixin; prediluted), *GAL* (sc‐166431, Santa Cruz; 1/50), and *MMP3* (MAB905, R&D; 1/20). Sections were then incubated with the two‐step Polymer Detection System (Polink‐2 Plus, GBI, USA), and detection was performed with the Dako Envision System using diaminobenzidine. Specimens were then lightly counterstained with Mayer's hematoxylin, dehydrated, and mounted. Negative controls were obtained by replacing the specific primary antibody with animal serum. A positive control sample was evaluated with each batch of slides.

IHC results were scored by two experienced pathologists who were blind to clinical and follow‐up information. Protein expression was determined based on staining intensity and area. The staining intensity was graded as 0 (no staining), 1 (weak staining), 2 (moderate staining), or 3 (strong staining). The percentage of immunoreactive cells was graded as 0 (≤10%), 1 (11%‐25%), 2 (26%‐50%), 3 (51%‐75%), and 4 (>75%). The IHC score was calculated by multiplying the intensity and the percentage of positive tumor cells. Samples with IHC scores ≥3 were designated as positive, and samples with IHC scores <3 were designated as negative (16).

### 2.3 Statistical analysis

To compare the differences in demographic and clinical factors between the two independent cohorts, Student's *t* test or Mann‐Whitney test was used for continuous variables, and Chi‐square test was used for categorical variables. OS was defined as the time from surgery to death resulting from any cause, which was estimated by the Kaplan‐Meier method. Differences between survival curves were examined using the log‐rank test. Multivariate survival analysis was performed using the Cox proportional hazards model. Receiver operating characteristic (ROC) curves were plotted to assess the area under the curve (AUC) with a 95% confidence interval (CI).

A nomogram was formulated based on the prognostic factors with significant differences in the Kaplan‐Meier analysis of the entire cohort and delineated using the “rms” R package. The selection of the final model was performed using a backward step‐down process with the Akaike information criterion. All tests were two‐sided, and statistically significant results were determined as *P* < .05. Statistical analyses were performed by SPSS software (version 18.0), GraphPad Prism (version 5), MedCalc (version 9.6.2.0), or R software (version 3.2.3).

## 3 RESULTS

### 3.1 Gene selection

The screening strategy for ESCC prognosis‐associated genes has been previously described.^17^ In brief, recurrent somatic CNVs with high frequency (defined as more than three samples) were screened using whole‐exome sequencing data from 81 ESCC samples,^9^ and exome sequencing data files are available at the European Genome‐phenome Archive (EGA), under accession EGAS00001000932. Amplifications and deletions with stringent thresholds were defined as fold change ≥3.0 for amplification and <0.25 for deletion. Then, 76 CNVs were selected for further analysis.

**Figure 1:**
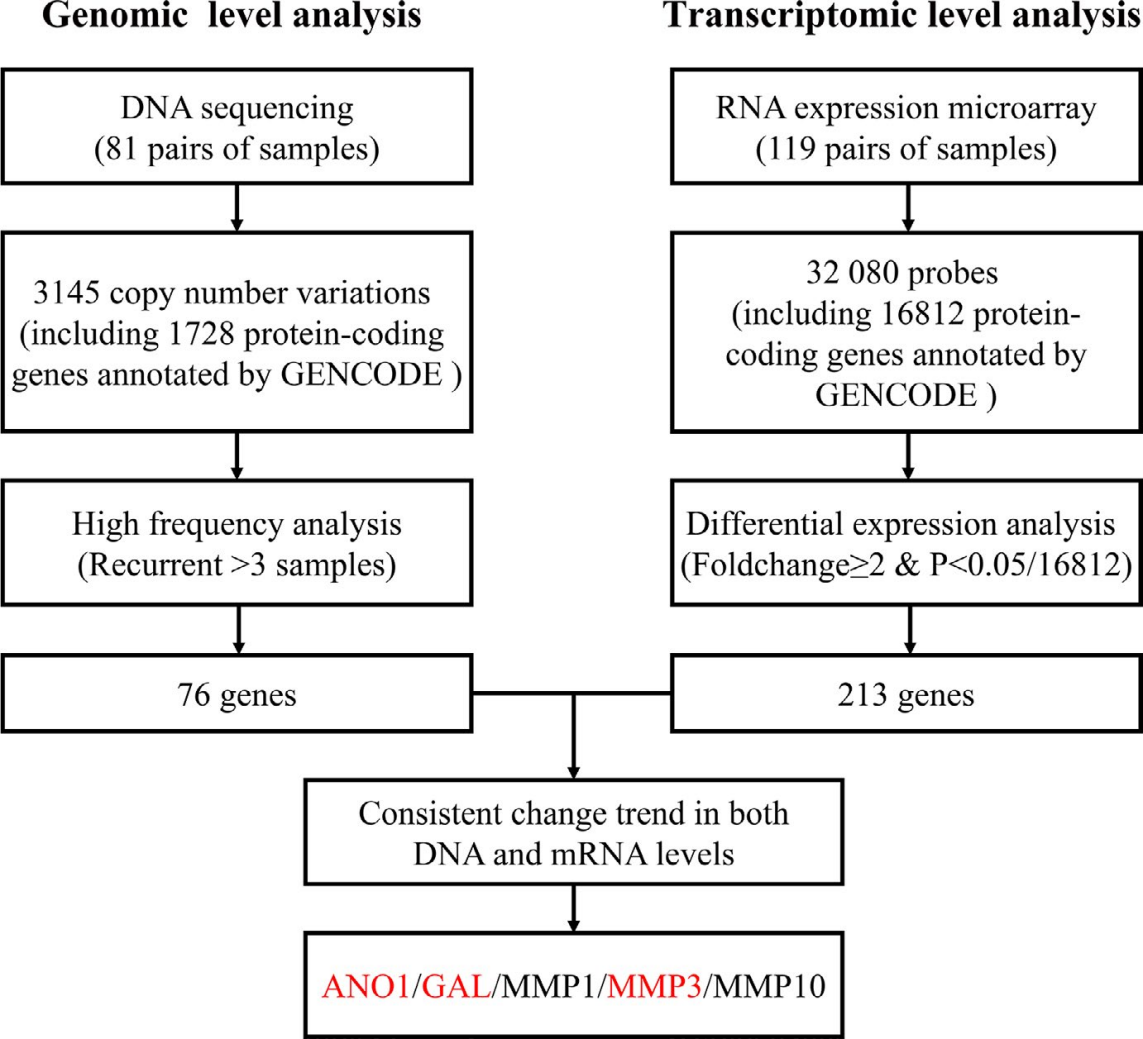
Flowchart of selecting the immunohistochemistry panel for prognostic evaluation in esophageal squamous cell cancer

In addition, we performed transcriptomic level analysis with gene expression microarrays on 119 paired ESCC tumor and adjacent normal tissues.^16^ Transcriptomic microarray data files are available at the Gene Expression Omnibus (GEO) Database, under accession http://www.ncbi.nlm.nih.gov/geo/query/acc.cgi?acc=GSE53624 (ID: 200053624). Of the 32 080 probes in the microarray, 16 812 genes, which were annotated as protein‐coding genes in Gencode v19, were initially reserved in the analysis. Then, 213 mRNAs with a fold change ≥2 and *P < *2.97 × 10^−6^ (0.05/16812, Bonferroni correction) were defined as differently expressed for further analysis. The integrated analysis of 76 CNVs and 213 differently expressed mRNA indicated that five genes (*ANO1*, *GAL*, *MMP1*, *MMP3,* and *MMP10*) showed consistent changes at both genomic and transcriptomic profiles (Figure 1). Notably, *MMP1*, *MMP3,* and *MMP10* belong to the *MMP* family and have similar biological function. In view of the congruent changes in the mRNA expression of *MMP1*, *MMP3,* and *MMP10*, we selected *MMP3* as the representative of these three genes because of its maximum overexpression in mRNA level. Thus, three genes, namely, *ANO1*, *GAL,* and *MMP3,* were kept in the final list for additional IHC tests.

**Figure 2:**
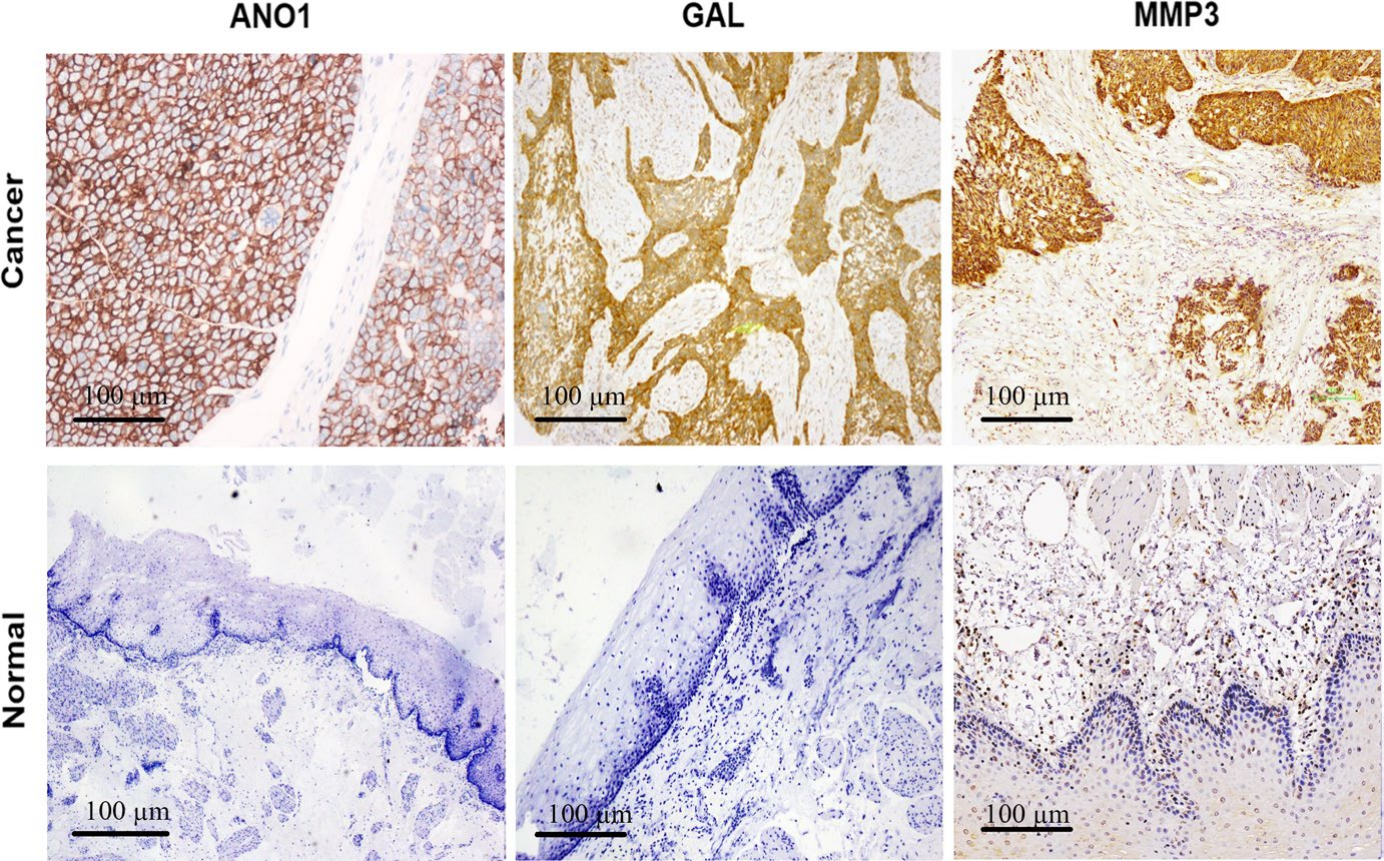
Representative images of immunohistochemical staining of *ANO1*, *GAL,* and *MMP3* proteins in paired esophageal squamous cell carcinoma and normal adjacent tissues (100×)

Representative IHC images of three genes in ESCC and paired normal tissues are shown in Figure 2. *ANO1* protein was strongly stained on the cell membrane in tumor tissues. *GAL* protein was stained on the cell membrane and cytoplasm in tumor tissues, and *MMP3* protein was stained on the cell cytoplasm in tumor tissues. *ANO1* protein stained positive in 19.8% (39/197) tumor tissues and 1% (2/197) normal tissues. *GAL* protein stained positive in 58.9% (116/197) tumor tissues and 2.5% (5/197) normal tissues. *MMP*3 protein stained positive in 32% (63/197) tumor tissues and 3% (6/197) normal tissues. The positive expression rates of *ANO1*, *GAL,* and *MMP3* in ESCC tumor tissues were significantly higher compared to those in normal tissues (all *P* at <.001).

### 3.2 Survival analysis in the training group

The 5‐year OS was 42% for the training group (CAMS set), and the median follow‐up time of 197 patients was 34 months (1‐84.4 months). Univariate survival analysis revealed that *ANO1* (*P* = .015) and *MMP3* (*P* < .001) showed prognostic significance for all patients in the training group. However, *GAL* was not a prognostic factor (*P* = .091; Table 1). Other potential clinical covariates were also tested for their relationships with clinical outcomes of ESCC patients. Age (*P* = .007), N classification (*P* < .001) and differentiation (*P* = .027) were statistically significant predictors of the OS in the univariate analysis (Table 1).

**Table 1:** Univariate and multivariate survival analysis of clinicopathological characteristics, and expression of immunohistochemical markers in training and validation sets

Factors	Training set						Validation set					
	No. (%)	5‐y OS	Univariate		Multivariate		No. (%)	5‐y OS	Univariate		Multivariate	
			*P*	HR (95% CI)	*P*	HR (95% CI)			*P*	HR (95% CI)	*P*	HR (95% CI)
Sex			.655	1.11 (0.71‐1.75)	/	/			.648	1.12 (0.69‐1.79)	/	/
Male	160 (81.2)	43.1%					76 (64.4)	39.4%				
Female	37 (18.8)	36.5%					42 (35.6)	35.7%				
Age			.007	1.65 (1.15‐2.38)	.026	1.50 (1.04‐2.18)			.882	1.04 (0.65‐1.64)	/	/
≤60 y	106 (53.8)	48.8%					56 (47.5)	39.2%				
>60 y	91 (46.2)	34%					62 (52.5)	37.1%				
T classification			.184	1.39 (0.86‐2.25)	/	/			.046	1.93 (1.01‐3.77)	.508	1.27 (0.63‐2.54)
T_1‐2_	40 (20.3)	51.1%					23 (19.5)	56.5%				
T_3‐4_	157 (79.7)	39.4%					95 (80.5)	33.6%				
N classification			<.001	2.09 (1.41‐3.09)	.003	1.85 (1.24‐2.74)			<.001	2.73 (1.72‐4.35)	<.001	2.81 (1.71‐4.62)
N_+_	111 (56.3)	29.4%					49 (41.5)	52.2%				
N_0_	86 (43.7)	58%					69 (58.5)	18.1%				
Differentiation			.027	1.38 (1.04‐1.84)	.296	1.17 (0.87‐1.56)			.189	1.26 (0.87‐1.84)	/	/
High	35 (17.8)	49.1%					46 (39)	41.3%				
Middle	110 (55.8)	44.5%					60 (50.8)	38.3%				
Low	52 (26.4)	30.8%					12 (10.2)	25%				
Location			.082	0.79 (0.61‐1.03)	/	/			.365	0.86 (0.56‐1.31)	/	/
Upper	25 (12.7)	25.6%					5 (4.2)	20%				
Middle	81 (41.1)	40.7%					30 (25.4)	40%				
Lower	91 (46.2)	47.3%					83 (70.3)	38.5%				
ANO1			.015	1.69 (1.11‐2.57)	.003	1.93 (1.25‐2.97)			.017	1.93 (1.11‐3.37)	.004	2.42 (1.33‐4.39)
Negative	158 (80.2)	45.9%					96 (81.4)	40.6%				
Positive	39 (19.8)	25.6%					22 (18.6)	27.3%				
GAL			.091	1.39 (0.95‐2.02)	/	/			/	/	/	/
Negative	81 (41.1)	48.6%					/	/				
Positive	116 (58.9)	37.1%					/	/				
MMP3			<.001	2.09 (1.45‐3.03)	<.001	2.12 (1.44‐3.11)			<.001	3.73 (2.32‐5.99)	<.001	5.04 (3.01‐8.45)
Negative	134 (68)	50.4%					82 (69.5)	48.7%				
Positive	63 (42)	23.8%					36 (30.5)	13.9%				

The significant predictors of OS determined in the univariate analysis were further analyzed using Cox multivariate regression, and the final models showed that age, N classification, *ANO1*, and *MMP3* were independent predictors of OS in patients with ESCC in the training cohort (Table 1).

**Figure 3:**
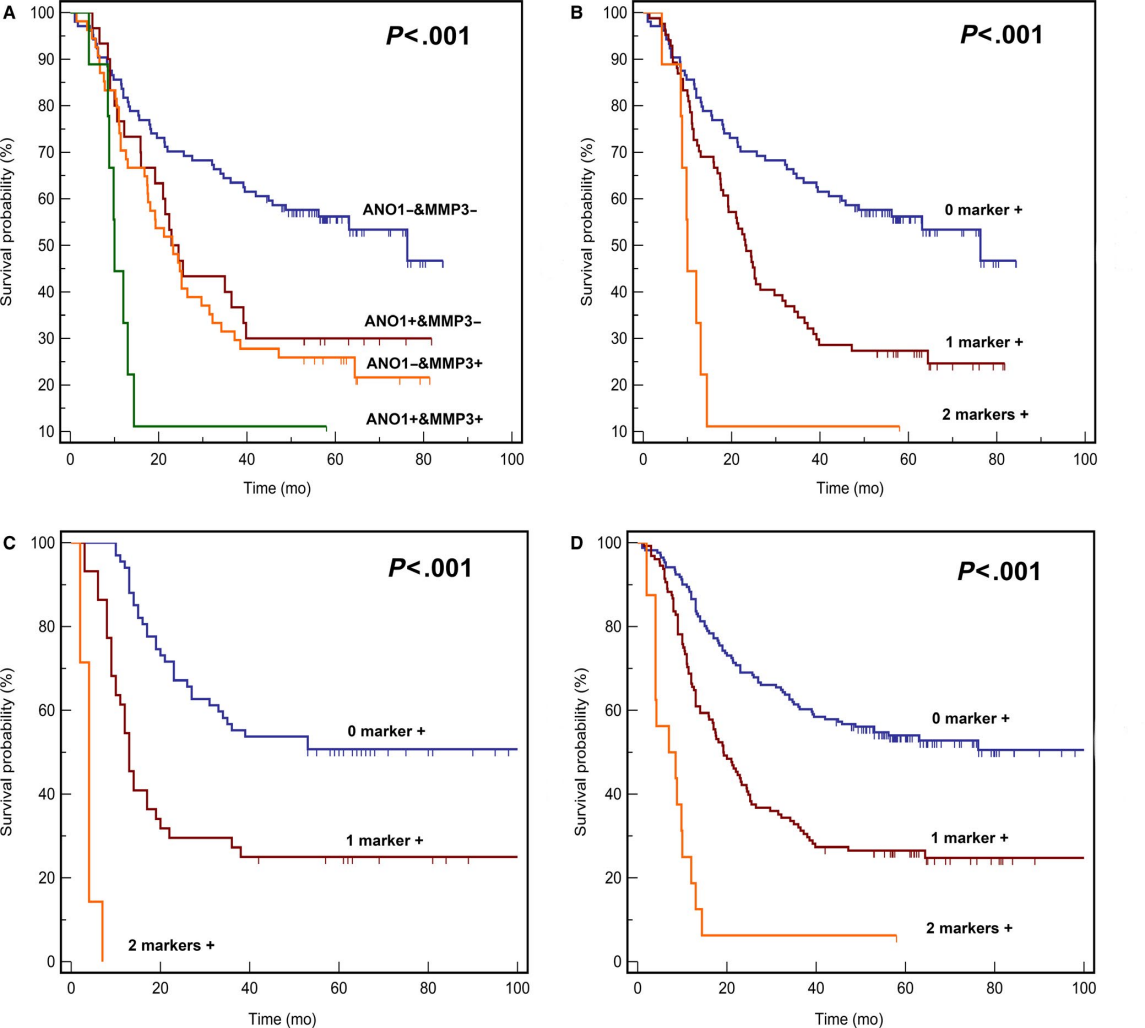
A, Application of the immunohistochemistry (IHC) panel to the training cohort segregated patients into different prognostic groups (*P* < .001). B, Application of the modified IHC panel to the training cohort segregated patients into three main prognostic groups (*P* < .001). C, Application of the modified IHC panel to the validating cohort segregated patients into three main prognostic groups (*P* < .001). D, Application of the modified IHC panel to the entire cohort segregated patients into three main prognostic groups (*P* < .001)

The training group was divided into four subgroups (*ANO1*−/*MMP3*−, *ANO1*+/*MMP3*−, *ANO1*−/*MMP3*+*,* and *ANO1*+/*MMP3*+) based on the expression status of *ANO1* and *MMP3* in ESCC tumor tissues. Kaplan‐Meier analysis revealed that 5‐year survival rates for those with *ANO1*−/*MMP3*−, *ANO1*−/*MMP3*+, *ANO1*+/*MMP3*−, and *ANO1*+/*MMP3*+ were 56.4%, 30%, 25.9%, and 11.1%, respectively (*P* < .001, Figure 3A).

When patients in the training cohorts were grouped according to the total number of positive IHC markers, there was no difference in the OS between patients with *ANO1*−/*MMP3*+ and those with *ANO1*+/*MMP3*− (5‐year OS, 30% vs 25.9%, respectively; *P* = .542). Therefore, the two groups were combined. As shown in Figure 3B, patients with two positive IHC markers had a much poorer prognosis compared with patients with one or zero positive marker (5‐year OS, 11.1% vs 27.4% vs 56.4%, respectively; *P* < .001).

### 3.3 Validating prognostic value of the two biomarker IHC panel

Further validation of the prognostic value of the IHC panel was performed in an independent cohort from another center in South China at the First Affiliated Hospital of Nanjing Medical University/Jiangsu Province Hospital (JSPH set). The 5‐year OS of these 118 patients was 38.1%, and the median follow‐up time of the validation group was 23 months (2‐170 months). As shown in Figure 3C, patients were stratified into three subgroups with different risks based on the positive marker status of two genes, and the 5‐year OS of each subgroup for each additional positive marker decreased by 25%.

We integrated the CAMS set and JSPH set as one cohort to validate the IHC panel. As shown in Figure 3D and Table 2, the prognostic IHC panel, age, T classification, N classification, and differentiation were identified as significant prognostic factors in univariable analysis. Multivariate analysis showed that age, T classification, N classification, and IHC‐based classifier remained independent predictors for OS of ESCC patients (Table 2).

**Table 2:** Univariate and multivariate survival analysis of clinicopathological characteristics, and immunohistochemistry (IHC) panel in all patients

Factors	No. (%)	5‐year OS	Univariate			Multivariate^a^		
			*P*	HR	95% CI	*P*	HR	95% CI
Sex			.482	1.12	0.81‐1.55	/	/	/
Male	236	41.8%						
Female	79	36.5%						
Age			.023	1.39	1.04‐1.85	.009	1.47	1.11‐1.96
≤60 y	162	45.4%						
>60 y	153	35.2%						
T classification			.018	1.59	1.08‐2.35	.02	1.59	1.08‐2.37
T_1‐2_	63	53.5%						
T_3‐4_	252	37.2%						
N classification			<.001	2.22	1.65‐2.99	<.001	2.06	1.53‐2.79
N_+_	155	55.4%						
N_0_	160	25.9%						
Differentiation			.018	1.28	1.03‐1.59	.439	1.09	0.88‐1.35
High	81	45.1%						
Middle	170	42.2%						
Low	64	28.7%						
Location			.091	0.84	0.68‐1.04	/	/	/
Upper	30	25%						
Middle	111	40.5%						
Lower	174	42.9%						
IHC panel			<.001	1.85	1.46‐2.34	<.001	1.81	1.42‐2.30
0	194	49.8%						
1	110	26.4%						
2	11	18.2%						

a Variables that showed significant association with esophageal squamous cell carcinoma (ESCC) prognosis were included in the regression analysis.

### 3.4 Prediction accuracy of the two biomarker IHC panel

**Figure 4:**
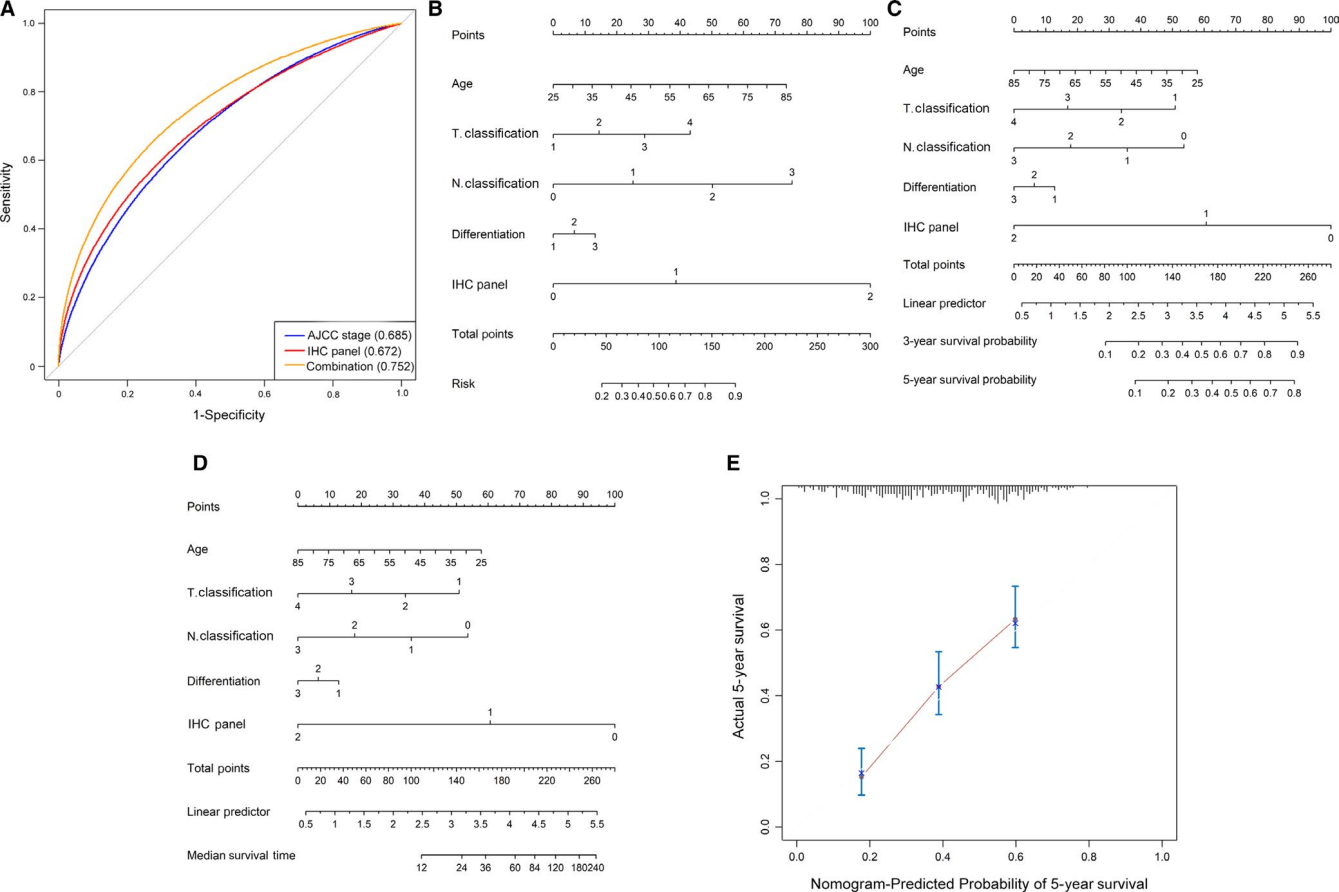
Receiver operating characteristic (ROC) curve analysis and nomogram. A, ROC curve analysis compares the prognostic value of the immunohistochemistry (IHC) panel with American Joint Commission on Cancer (AJCC) staging. B‐D, Nomogram integrating IHC markers and clinicopathological factors. E, Evaluation of the nomogram using 5‐year nomogram calibration curves. The dashed line represents an ideal evaluation, whereas the red line represents the performance of the nomogram. Differentiation: 1 = high, 2 = middle, 3 = low

All 315 patients in both the training and validation cohorts were then designated as good prognosis (128 patients) or poor prognosis (187 patients) based on if the survival time was longer than 5 years. Receiver operating characteristic curves were plotted to determine the prognostic predictive efficiency of the IHC prognostic panel for ESCC, and the results showed that the AUC was 0.672 with 95% CI from 0.619 to 0.724. The 8th AJCC staging was also analyzed, and the AUC was 0.685 with 95% CI from 0.631 to 0.74. Further analysis found that there was no significant difference between the two ROC curves of the IHC panel and AJCC staging (*P* = .717). The combination of the IHC panel and eighth AJCC staging yielded a better prognostic predictive efficacy for patients with ESCC (AUC: 0.752, 95% CI: 0.700‐0.805), which was superior to that of the two individual indexes alone (*P* < .001; Figure 4A). The similar results were observed in both the training and validation sets (Table 3).

**Table 3:** Receiver operating characteristic curve analysis compares the prognostic value of immunohistochemistry (IHC) panel with the eighth American Joint Commission on Cancer (AJCC) staging

Groups	Factors	AUC	95% CI	*P*_AUC_
Training set (CASM set)	AJCC staging	0.687	0.617‐0.757	Ref.
	IHC panel	0.668	0.600‐0.736	.705
	Combination	0.751	0.684‐0.818	.015
Validation set (JSPH set)	AJCC staging	0.697	0.613‐0.781	Ref.
	IHC panel	0.679	0.596‐0.761	.757
	Combination	0.770	0.689‐0.851	.031
Entire cohort	AJCC staging	0.685	0.631‐0.74	Ref.
	IHC panel	0.672	0.619‐0.724	.717
	Combination	0.752	0.700‐0.805	<.001

### 3.5 Nomogram building and its clinical utility

To provide a clinically useful tool to predict prognosis, we constructed a nomogram by integrating the IHC panel and multiple ESCC prognostic factors with significant differences in the Kaplan‐Meier analysis, including age, T classification, N classification, differentiation, and IHC panel (Figure 4B‐D). Calibration curves showed good performance of the nomogram with high consistency between the 3‐ or 5‐year OS estimates from the nomogram and those derived from Kaplan‐Meier estimates. Decision curve analysis was used to evaluate the potential of clinical application of the IHC‐based nomogram by quantifying the net benefits (Figure 4E). For predictive accuracy of OS, the bias‐corrected C‐index of the nomogram was 0.695 with a 95% CI of 0.657‐0.734.

## 4 DISCUSSION

ESCC is a clinically heterogeneous disease. The traditional AJCC staging system based on clinical features is a valuable tool for predicting prognosis and guiding treatment, but the system also has some deficiencies. Patients with the similar AJCC stage may have different outcomes (18), and the differences in prognosis might be attributed to biological heterogeneity. Therefore, it is urgent to discover novel molecular biomarkers for ESCC prognosis. Assignment of prognosis based on tumor molecular characteristics is an increasingly promising approach. Great efforts have been made to search for the molecular biomarkers of ESCC from mRNA, long noncoding RNA, and microRNA to protein biomarkers.^16^, ^18^, ^19^, ^20^ However, most of these markers have limited detection ability and have not been adopted for clinical application. For example, some of these biomarkers were screened through candidate or pathway‐based strategies rather than systematic screening, which leads to limited effectiveness in prediction.^19^, ^21^, ^22^ In addition, for most biomarkers, the underlying biological mechanisms by which these biomarkers influence the progression of ESCC are not well understood.^18^, ^21^, ^22^ Moreover, the relatively small sample size and the deficiency of independent validation in homologous populations may restrict the reliability and utility of biomarkers.^18^, ^21^, ^23^ Therefore, a systematic review on IHC prognostic markers of ESCC was performed by He et al in 2017, they screened the retrieved literature and found eight markers, such as *EGFR*, *p‐mTOR*, *Cyclin D1*, *Survivin*, *VEGF*, *Podoplanin*, *Fascin,* and *PKM2* indicating unfavorable prognosis and three markers (*P27*, *P16,* and *E‐cadherin*) indicating favorable prognosis of ESCC.^24^ These IHC prognostic markers of ESCC are involved in regulating proliferation, cell apoptosis, angiogenesis, invasion, and metastasis of ESCC cell as reported in original studies. The valuable systematic review identified several IHC prognostic markers in ESCC; combination of these prognostic markers as a panel may be a useful tool for improving predicted accuracy, a large prospective clinical trial is needed.

In contrast to prior studies, we screened candidate prognostic markers based on an integrated analysis of genomic and transcriptomic profiles from our previous studies in Chinese population. By this novel method, we hoped to find new markers for prognostic evaluation and therapeutic target in ESCC. Finally, we identified two genes (*MMP3* and *ANO1*) associated with the prognosis of ESCC patients.

*ANO1* is located on chromosome 11q13, and amplification of 11q13 is a common event in cancers from multiple anatomical sites.^25^, ^26^ *ANO1* is upregulated and correlates with poor prognosis in several cancers.^27^, ^28^, ^29^ A previous study has also found that positive *ANO1* is a promising biomarker to predict the unfavorable outcome for ESCC patients even in precancerous lesions.^30^ Our high‐throughput data showed that *ANO1* expression was significantly upregulated in ESCC tumor tissues at both mRNA and protein levels. Moreover, patients with a positive expression of *ANO1* had a poorer prognosis, suggesting that *ANO1* may contribute to tumorigenesis of ESCC. In our previous study, we found that *ANO1* promotes ESCC cell proliferation, migration, and invasion by activating the *TGF‐β* pathway, suggesting that *ANO1* is a novel oncogene and may serve as a potential therapeutic target in ESCC.^17^

Matrix metalloproteinases (*MMPs*) are multifunctional zinc‐dependent proteinases that play a fundamental role in the physiological degradation of the extracellular matrix in angiogenesis, tissue repair, and tissue morphogenesis.^31^ Previous studies have shown that *MMPs* play a critical role in the invasion and metastasis of most malignancies, especially in ESCC.^32^ This study found that several genes in the *MMP* family were significantly upregulated in the expression microarray data, including *MMP* 1, 3, 8, 10, and 12, and *MMP3* was selected as the representative for the IHC study because it was the most upregulated MMP gene in the expression microarray. Furthermore, the IHC results also showed that *MMP3* was a significant predictor for the prognosis of ESCC.

We next combined *ANO1* and *MMP3* as a prognostic IHC classifier. Our results suggested that the IHC panel consisting of *ANO1* and *MMP3* can be used as an independent prognostic predictor of ESCC and can divide ESCC patients into three different risk subgroups based on zero, one or two positive markers. The 5‐year OS for each additional positive marker decreased by 25% in the validation cohort.

Further ROC analysis was performed to compare the prognostic predictive efficiency of the two marker IHC panel with current staging systems. The results showed that there was no significant difference between the two ROC curves of the IHC panel and AJCC staging, but the combination of the two biomarker IHC panel and the 8th AJCC staging yielded a better prognostic predictive efficacy for patients with ESCC. Therefore, the two biomarker IHC panel provides clinicians with a valid and reliable tool for better prediction of ESCC prognosis and can be an outstanding supplemental tool with AJCC staging for evaluating the prognosis of ESCC patients. In addition, the IHC panel and the clinicopathological variables of poor prognostic features, including age, T classification, N classification, and differentiation, were integrated into a prognostic nomogram. Calibration plots revealed a good correlation between the predicted survival probability and the actual survival, which showed high potential of clinical application of the nomogram.

In conclusion, we performed an integrated analysis using the genomic and transcriptomic profiles from ESCC samples. Two prognostic biomarkers (*ANO1* and *MMP3*) were identified, and a valuable prognostic model was constructed to predict the outcome of ESCC patients. Compared with the traditional TNM stage system, this model showed a better prediction efficiency. This is the first report to describe a two marker IHC panel that includes *ANO1* and *MMP3* that can be used to assess the prognosis of ESCC. However, our study also had some limitations. Although we selected candidate genes based on the CNVs and expression levels of mRNA, other factors may influence protein expression, including epigenetic changes, transcriptional control, and posttranslational modification. Further prospective, multicenter studies with larger sample sizes are required to validate the clinical value of the two biomarker prognostic panel in ESCC patients.

## CONFLICT OF INTEREST

The authors have no conflict of interest.

## Data Availability

I confirm that my article contains a Data Availability Statement even if no data is available (list of sample statements) unless my article type does not require one. I confirm that I have included a citation for available data in my references section, unless my article type is exempt.
